# Motherese by Eye and Ear: Infants Perceive Visual Prosody in Point-Line Displays of Talking Heads

**DOI:** 10.1371/journal.pone.0111467

**Published:** 2014-10-29

**Authors:** Christine Kitamura, Bahia Guellaï, Jeesun Kim

**Affiliations:** 1 School of Social Science and Psychology, University of Western Sydney, Sydney, Australia; 2 MARCS Institute, University of Western Sydney, Sydney, Australia; 3 Laboratoire Ethologie, Cognition, Développement, Université Paris Ouest Nanterre La Défense, Paris, France; University of Tuebingen Medical School, Germany

## Abstract

Infant-directed (ID) speech provides exaggerated auditory and visual prosodic cues. Here we investigated if infants were sensitive to the match between the auditory and visual correlates of ID speech prosody. We presented 8-month-old infants with two silent line-joined point-light displays of faces speaking different ID sentences, and a single vocal-only sentence matched to one of the displays. Infants looked longer to the matched than mismatched visual signal when full-spectrum speech was presented; and when the vocal signals contained speech low-pass filtered at 400 Hz. When the visual display was separated into rigid (head only) and non-rigid (face only) motion, the infants looked longer to the visual match in the rigid condition; and to the visual mismatch in the non-rigid condition. Overall, the results suggest 8-month-olds can extract information about the prosodic structure of speech from voice and head kinematics, and are sensitive to their match; and that they are less sensitive to the match between lip and voice information in connected speech.

## Introduction

Young infants need to make sense of an array of multisensory stimulation that pervades their everyday experience. This includes the mother's talking face usually positioned facing the infant ensuring that her speech is both seen and heard. It is well known that visual information influences the perception of speech. For adults, watching a talker's moving face assists them to understand speech in noisy [1] and quiet environments [2]. A growing body of research shows adults also use visual prosodic cues to stress, rhythm and intonation to enhance their understanding of expressive speech and they can do this without access to articulatory cues of the lips, jaw etc. [3], [4], [5]. These studies highlight the role peri-oral cues play in the perception of visual speech.

Much auditory-visual speech research concentrates on infants’ ability to relate phonetic information in the lips and the voice. Infants are typically tested facing side-by-side displays of two faces articulating two vowels, e.g.,/i/versus/a/, while hearing one vowel. They are deemed to detect congruency if they look longer to the matching side. Remarkably, there is evidence that from birth, infants detect equivalent phonetic information in the lips and voice [6] but auditory-visual phonetic matching is also shown at 2 months [7], at 4.5 months [8], [9], [10]; and at 8 months based on the gender of the talker [11]. However, it seems infants require the full spectral complexity in the voice to detect congruency, because when the vowels are reduced to, for example, sine-wave analogues or simple tones, they are no longer sensitive to audiovisual vowel correspondences [12]. Taken together, these studies suggest young infants already have the primitives of lip reading for single speech sounds.

At the syllable level, it is clear infants are sensitive to correspondences between lip and voice information. We are interested, however, in longer stretches of speech and whether infants can detect the prosodic commonalities in the multi-word utterances from their sight and sound. Evidence using continuous speech typically shows that young infants rely on the congruence between auditory emotions (happy, angry) and the appropriate facial gestures [13], [14]. Infants are also sensitive to temporal synchrony in continuous speech as they look longer to nursery rhymes that are presented with face and voice in synchrony than out of synchrony [15]. More recently, Blossom and Morgan examined sensitivity to the temporal synchrony of visual prosody using continuous infant-directed (ID) speech [16]. Similar to others, they found infants aged 10–11 months intermodally matched synchronous faces and voices, but in a second visual prosody only experiment (where the mouth was obscured) infants could not detect synchrony even when the delay was increased to 20 frames. A third experiment tested whether infants could match visual prosody (mouths obscured) when each of the two silent faces was speaking different passages. They found that with in-phase voices and faces, infants looked longer to the congruent face. This study indicates firstly, that visual prosody may be subject to different constraints than visual phonetic information, and secondly, that at 10–11 months, infants use visual prosody, especially as it is exaggerated in ID speech, to extract information about the structure of language.

Inherent in mother infant interactions is the exaggerated prosodic characteristics found in the mother's speech [17], [18]. The perceptual salience of ID over adult-directed (AD) speech has been well established in the auditory domain [19], [20] with its exaggerated prosody assisting infants in many of the challenges of early speech perception, e.g., to segment the speech stream [21], to find stressed words in sentences [22] to discriminate multi-syllabic words [23], and parse clauses [24]. ID speech is less studied in the visual domain, but does contain similar exaggerations to facial expressions and articulatory lip gestures for corner vowels [25], [26], [27].

**Figure 1:**
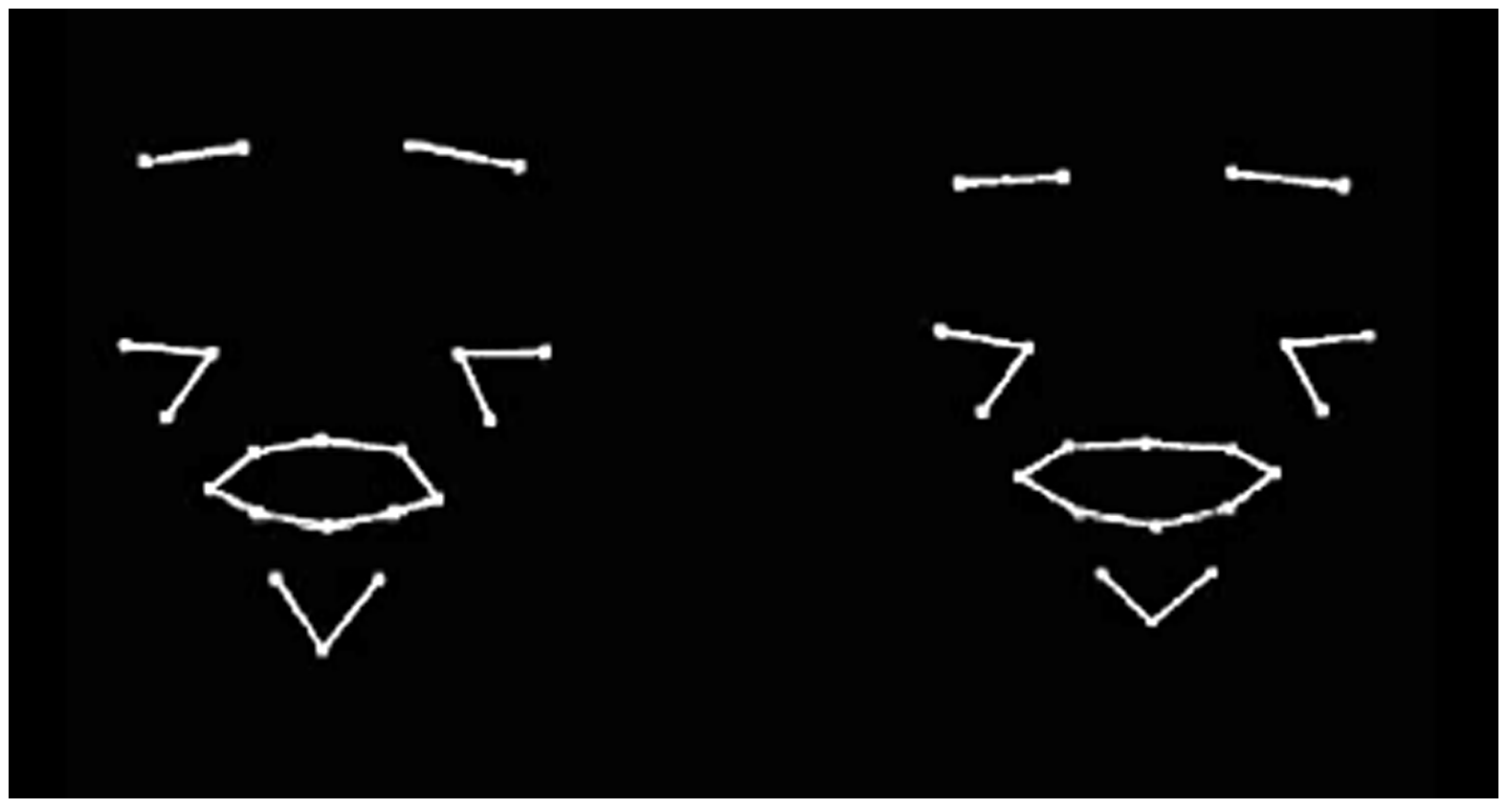
A static image of the side-by-side the point-line talking faces presented to the infants.

The aim of the present study is to examine whether infants are able to intermodally match multi-syllabic ID utterances from their auditory and visual signals. Evidence that the perceptual salience of ID speech prosody translates to the visual domain would suggest that infants’ speech perception is assisted by not only auditory but also visual speech prosody. The current study follows in the same vein to Blossom and Morgan, but we tested younger infants, 8-month-olds, on their ability to match the intermodal properties of auditory and visual prosody. As the visual counterpart, we used specially constructed line-joined point-light displays derived from the head and face motion of three females recorded reading short sentences to their babies. We used point-*line* rather than point-*light* displays because we were concerned that point-light displays might not provide infants with enough coherent information given the relatively sparse number of motion detectors used in our displays (see Figure 1). It is also important to note that placement of the mouth markers on the outer edges of the lips de-emphasizes the contrastive details of consonant articulation and tends to emphasize syllabic modulations. In other words, the lip displays do not provide fine articulatory detail. Our main objective was to test whether infants would be able to perceive the prosodic relations of auditory and visual speech by using stimuli that have been tailored to provide relatively more suprasegmental than segmental speech cues.

Importantly, these kinds of point-light displays permit face and head kinematics to be presented without other potentially distracting information such as form, texture, and other features. Point-light displays have been used in the past to show that infants can, for example, categorize displays of animals and vehicles [28]; extract emotional information [13] and recognise biological motion [29], [30], [31] but not to examine visual speech motion. Another benefit of using point-line displays derived from Optotrak data is that the motion produced during speech can be separated into rigid and non-rigid motion. Rigid motion refers to movements in which the head translates up-and-down, side-to-side, front-to-back and rotates (pitch, yaw, roll) whereas non-rigid motion refers to face-internal movements that change the spatial configuration of the facial surface over time, e.g., articulatory gestures of the lips and jaw, and expressions provided by eye and cheek region.

The auditory-visual stimuli were developed taking into account properties to which infants are sensitive, that is, the global prosodic motion signaled by rigid (head) movement, and the modulation of locally produced syllables in non-rigid motion (face internal). For the mouth movement, we were trying to match the “grain-size” of information across the two modalities. For example, in auditory perception infants pay particular attention to the syllable [32], [33], [34]. The bias to syllables is consistent with infants’ predilection to attend to the periodic portions of the speech signal [35] which carries prosodic information. Here, we use the term visual prosody to refer to all aspects of speech production that are used to visually convey the acoustic correlates of prosody (e.g. stress, rhythm, intonation). This assumes that peri-oral cues are mainly used in the perception of visual prosody but even with lip movement, there is a certain rhythm to the cyclic gesturing of the mouth opening and closing to signal the onset and offset of syllables [36]. Here we call this syllabic rhythm with the caveat that this is not necessarily reflective of the rhythmic structure of the language as the stimuli consist of mainly single syllable ID sentences. Indeed, we used pairs of sentences that differed in the number of syllables but were matched for duration to allow infants to focus on syllable differences.

At some point in development infants should become sensitive, as adults are, to the sequencing of visual syllable-sized events, that is, events that occur in relatively quick succession and are conveyed by the rhythmic sequencing of syllabic information in the mouth region. In this study the sentences presented to each infant varied in the syllable difference between each pair of sentences (see Table 1). For infants, we categorize these events as occurring at a *local rhythmic* level because exaggerated mouth openings should act as a syllabic timing device before acting as a cue to phonetic grouping in an utterance [37]. Using sentence pairs with unequal syllables should make it easier for infants to match face and voice when the syllable difference between sentences is larger (see Table 1). This is especially pertinent if infants are paying attention to the information carried in non-rigid face internal motion. Over and above the local syllabic cues, infants should derive meaning from *global intonation*, that is, exaggerated intonational contours that extend over larger temporal windows. These larger intonational groupings are conveyed movements of the head (e.g. nodding and tilting) and unfold more slowly over time. Given a talking face/head produces auditory-visual signals that consist of multiple events that occur at different timescales, we expect infants to be more sensitive to global intonational groupings than to local rhythmic ones.

**Table 1:** Sentence pairs from female talker 1, 2 and 3 showing the sentence pairs (A and B) and the number of syllables in each sentence in parenthesis.

Female Talker	Sentence A (syllable no)	Sentence B (syllable no)	Syllable Diff	Duration (secs)
1	Sheep (8)	Apple (10)	2	4
2	Clams (9)	Mouse (12)	3	6
3	Sheep (8)	Mouse (12)	4	5

The difference in syllable number between A and B, and duration of sentence pairs is shown in the last two columns. The sentences were: “Did you know, Woolly is a sheep” (Sheep); “Yum, clams are round, small, soft and tasty” (Clams); “Look, the big red apple fell to the ground” (Apple) and “It's tea time, let's feed the white mouse some flower seeds” (Mouse).

In this study we conducted two experiments. The aim of Experiment 1 was to establish whether 8-month-olds could match a talking face and voice, when (i) the auditory speech signal contained the full spectrum of speech frequencies; and (ii) the auditory signal was low-pass filtered at 400 Hz to provide mainly intonation in the auditory signal. Here, our question was whether there would be a difference in the degree to which infants could match full-spectrum versus low-pass filtered intonation-only speech to a talking face/head. The aim of Experiment 2 was to determine whether infants could match auditory and visual displays when one group was presented with (i) rigid-only head motion only; and another group with (ii) non-rigid face-internal motion only. As already stated, we expected infants would find it easier to match a talking face and voice using rigid head than non-rigid face motion in an intermodal preference procedure.

## Experiment 1: Full Spectrum versus Intonation-Only Auditory Speech

In Experiment 1 we tested two groups of 8-month-old infants to examine the role visual prosody plays in speech perception. We chose this younger age, as it has been established that 10 to 11-month-olds can match the intermodal properties of auditory and visual prosody in continuous speech. Both groups saw side-by-side silent visual displays and heard the auditory counterpart of one of the displays. The auditory counterpart for the first infant group was the full speech spectrum of multi-word utterances; and the auditory counterpart for the second group of infants was an intonation version of the utterances (low-pass filtered at 400 Hz), that is, all segmental information above 400 Hz is removed from the auditory speech signal.

### Method and Materials

All research was approved by the Human Research Ethics Committee (HREC) at University of Western Sydney. In accordance with ethics approval, written and oral consent was obtained from all mothers in the study.

#### Participants

Forty infants participated in Experiment 1 **.** For the full-spectrum auditory speech group, the final sample consisted of twenty 8-month-old infants (*M*age = 34.5 weeks; *range = *33.0−37.6 weeks; 13 males). Only infants who looked to each side for at least 2 seconds in at least 4 trials, and paid attention to both visual displays for at least 50% of the test period were included in the analysis. On this criterion, 5 additional infants were excluded due to lack of attention. For the intonation-only auditory speech group, the final sample consisted of twenty 8-month-old infants (*M*age = 35 weeks; *range = *32.7−38.6 weeks; 12 males). Six additional infants were excluded because they looked less than 50% of the time. Parental reports indicated that all infants were born full-term, were healthy at the time of testing, had no history of chronic or acute ear infections, and passed newborn hearing screening.

#### Speech stimuli

The speech stimuli consisted of a three pairs of sentences, one pair from each of the three female talkers (see Table 1). The sentences were adapted from the IEEE list of phonetically balanced sentences [38], and were “Yum, clams are round, small, soft and tasty” (9 syllables); “Look, the big red apple fell to the ground” (10 syllables); “It's tea time, let's feed the white mouse some flower seeds” (12 syllables) and the utterance “Did you know, Woolly is a sheep” (8 syllables). Adaptations to facilitate a more infant-directed style to the sentences were for instance, adding the word “Yum” and “Look” to the beginning of the utterances. From the list of sentences for each of the mother, pairs of sentences were selected that matched in duration but differed in syllable number. For some of the original sentence pairs there were small differences in duration (<500 ms). In these situations, the longer utterance was slowed, and the shorter utterance made slightly faster using Adobe Premier. The sentences and syllable differences for each female are shown in Table 1.

In Experiment 1, the point-line displays remained unchanged for both groups of infants, but the auditory component of each sentence was either presented as full spectrum speech or was low-pass filtered at 400 Hz in Praat using a Hann filter. This removes auditory segmental information but leaves prosodic cues such as intonation, and duration relatively intact.

#### Speech Recording Procedure

Three females were recorded using a Northern Digital Optotrak 3020 system and a Sennheiser MKH416P48U-3 floor microphone. Each female was a mother who sat facing her infant during the recording session. She was instructed to speak a list of sentences as she would if she were reading to her infant at home. Optotrak markers were attached to the female’s face-internal features and to a head rig to measure rigid head movement. There were 21 non-rigid face internal markers on the eyebrows (4), cheeks (6), mouth (8) and jaw (3); and 3 markers on the head rig. Each frame of speech movement was extracted from the raw marker positions, and represented in terms of its displacement from the first frame. Data reduction was achieved by using principle components (PC) analysis; the PC’s coefficients were used to generate point-line visual speech motion. An animated schema of the face was generated by displaying the PC’s coefficients generated in Matlab using a marker visualization tool. Rendering was achieved in Togl and OpenGL [39], and each frame was written to an image file and compiled to a video sequence at 25 fps. This line-joined version of visual speech did not capture fine detail because the motion capture markers were placed on the outside of the lips and so closure was never complete (see Movies S1 and S2). Such a sparse representation was chosen to emphasize rhythmic speech motion and the perception of simple onsets and offsets. Nevertheless, the motion of the display did correlate with auditory properties as measured over a rendition. So for example, we measured how changes in speech amplitude (RMS energy) and mean F0 correlated with the rigid motion of the display (in the X and Y axes) and with the motion of the eyebrows, mouth (lip height & width) and jaw. For each of the sentences, at least one correlation coefficient was 0.45 or greater indicating that there was always a significant relationship between some of the auditory and visual features.

#### Testing Procedure

Each infant was tested with one of the three talkers' sentence pairs (see Table 1). Infants were tested sitting on their parents’ lap in a darkened sound-attenuated room facing three computer monitors - a left, right and middle screen placed on a shelf in front of them. The infant’s gaze was centered at the start of all trials with an attention-getting looming video presented on the middle screen. Because the visual stimuli were unfamiliar point-line displays, we initially played the infants four repetitions of one sentence on the left monitor, and four repetitions of the other sentence on the right monitor. In the test trials, the visual sentence displays were played either on the same side or the opposite side to presentation in the initial phase. There were six test trials in which the auditory-only target sentence played through a central speaker with the two silent point-line displays presented simultaneously on the left and right monitors. We counterbalanced the target sentence so that half the infants heard one sentence in the pair and the other half heard the other sentence in the pair. The target sentence was also counterbalanced so that it was displayed on the left or right side equally. Each of the six test trials lasted for seven repetitions of each sentence. A digital video camera placed midline facing the infant, was connected to a video monitor in the control room and used by the experimenter to judge the infant's head and eye movements (left, right or no monitor) in real time. The experimenter used a computer keyboard to input the timing of the infant’s looking behavior: The left arrow key was pressed when the infant looked to the left monitor; the right arrow key when the infant looked to the right monitor, and the down arrow key when the infant was fixating the central monitor between trials. No key was pressed when the infant looked elsewhere. The experimenter was blind to the experimental manipulations, including the side of the matching visual display. To diminish parental influence, the same utterances with all the silences removed were played to parents using a Panasonic portable CD player and AKG studio headphones.

### Results and Discussion

The extent to which infants fixated the matching visual display was used to index the degree to which 8-month-olds detected congruency between the auditory and visual speech signals. Due to each sentence pair (1, 2 and 3) having different durations (see Table 1), the dependent variable was fixation duration as a percentage of total test trial duration for each sentence pair. Assumptions of normality were tested and met, both Shapiro-Wilk and Kolmogorov-Smirnov tests proved non-significant (*p*s>0.1). For both the full-spectrum and intonation-only auditory speech groups, the preliminary ANOVAs testing the effects of the three counterbalancing variables, [(i) same or different side for initial trial and test trials, (ii) left or right presentation of visual target sentence and (iii) target auditory sentence 1 or 2 in pair] proved non-significant. We use partial eta^2^ to report effect size.

A 2 × 3 × ANOVA testing group (full-spectrum vs. intonation-only) and syllable difference (2, 3, or 4 syllables) between participants and trial (matching vs. mismatching) within participants revealed significant main effects for trial *F*(1,34) = 14.6, *p*<0.001; *η_p_^2^* = 0.3; groups *F*(1,34) = 17.8, *p*<0.0001; *η_p_^2^* = 0.3 and syllable number *F*(1,34) = 3.9, *p*<0.03, *η_p_^2^* = 0.19. There were no significant 2- or 3-way interactions (*p*s>0.23). Raw looking time scores (seconds) were also analyzed (ANOVA) with the same main effects and interactions. Irrespective of whether infants heard full-spectrum or intonation-only speech, 8-month-olds looked significantly longer to the matching than mismatching display. In addition, infants found low-pass filtered speech more interesting than full-spectrum speech in the context of this task. Thus, despite the preference for matching displays by both groups, overall percentage looking time in the intonation-only group (80%) was greater than in the full-spectrum group (61%).

The main effect for syllable difference showed that, irrespective of visual preference, infants looked less overall in the 3-syllable condition than the 2- and 4-syllable conditions. No firm conclusions can be drawn from this finding, as syllable difference was conflated with female talker, that is, the female talker who had the lowest percentage looking time was also the talker who displayed the least motion when talking to her infant.

**Figure 2:**
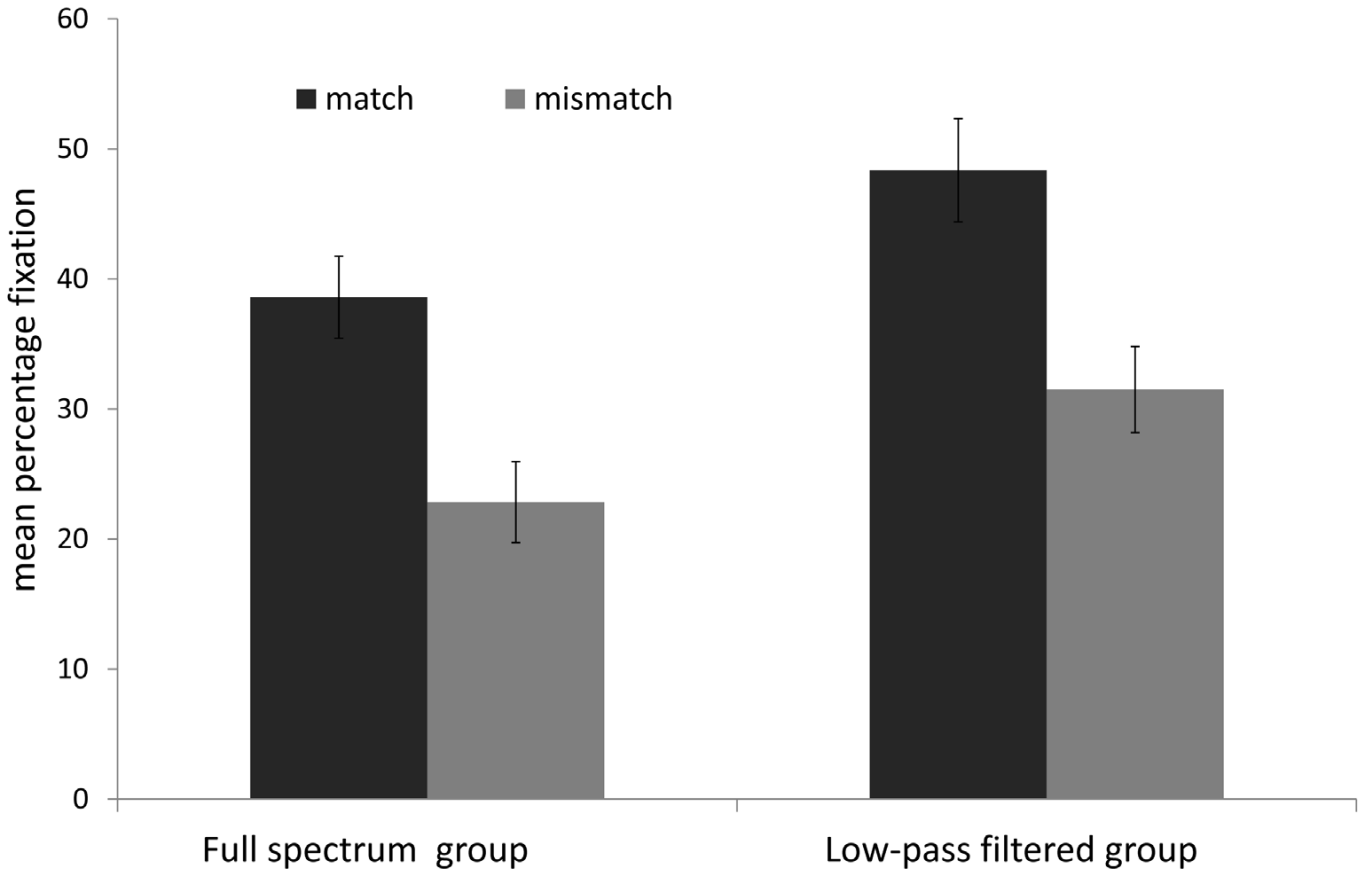
Mean percentage fixation to the matching and mismatching point-line displays for the full spectrum and prosody-only groups in Experiment 1. Error bars = 1 standard error of the mean.

Importantly, infants spent a greater percentage of trial duration looking to the matching (*M* = 38.6%) than the mismatching display (*M* = 22.8%) in the full spectrum group and to the matching (*M* = 48.4%) than mismatching display (*M* = 31.5%) in the intonation-only group (see Figure 2). Of the 20 infants in the full-spectrum group, 17 infants looked longer at the matching than mismatching point-line display (*binomial p* = .001). In the intonation-only group, 15 of the 20 infants, looked longer at the matching than mismatching visual display (*binomial p* = .01).

In sum, the results demonstrate that under conditions in which prosody is exaggerated in both auditory and visual domains, infants have no difficulty perceiving congruent temporal and spatial speech patterns across modalities, even when the visual signal consists only of animated markers of a talking face, and the speech is low-pass filtered. That infants perceive congruence even without the benefit of fine-grain phonetic information in the visual and/or auditory displays, suggests that the AV match occurred at the prosodic level (e.g., phrasal intonation and/or syllabic rhythm). Here, it should be noted that the filtered auditory speech maintained prosodic information such as intonation, syllable rhythm, word stress etc. The degraded visual speech also included information in mouth/jaw movements at a local rhythmic level, and other global prosodic groupings in the head movements. So, it is not clear from these results whether it is global prosodic movement in the head or local syllabic rhythm from the mouth that is recognized and matched across modalities. It is possible that coarse grain articulatory information was recovered from the mouth/jaw motion. To resolve this issue, it is necessary to separate this information in the visual domain, and examine whether infants can match auditory and visual speech signals.

## Experiment 2: Rigid versus Non-Rigid Motion

Experiment 2 presented one group of infants with (i) rigid-only head motion only holding non-rigid motion constant; and another group with (ii) non-rigid face-internal motion only holding rigid motion constant. The aim was to examine separately, the contribution that (i) global prosody and (ii) the local property of syllable sequencing may make to the infant's ability to match auditory and visual connected speech. The rigid-only motion condition tested whether infants could detect the congruence between global prosodic properties, such as intonation, word stress and/or phrasal rhythm conveyed by head motion and the voice. The non-rigid condition, on the other hand, tested whether infants were sensitive to the temporal sequencing of the seen and heard syllables found in the non-rigid movements of the mouth/jaw. The effect of temporal synchrony is powerful, as it coordinates both temporal and spatial aspects of seen and heard speech events, and evidence shows that infants detect the congruence between lip movements/shape and speech sounds at the syllable level [8], [40]; and have a proclivity for the synchrony between voice and lips in longer stretches of speech [15].

### Method and Materials

#### Participants

The final sample consisted of 40 8-month-old infants. None of these infants participated in Experiment 1. In the rigid-only condition there were 20 infants (*M*age = 34 weeks; *range = *32.3–36.8 weeks; 9 males). Six additional infants were excluded due to lack of attention (4) and fixation to one side (2). In the non-rigid condition, there were 20 infants (*M*age = 34.4 weeks; *range = *33.1−38 weeks; 12 males). Six additional infants were excluded due to lack of attention (5) and mother interference (1). Parental reports indicated that all infants were born full-term, were healthy at the time of testing, had no history of chronic or acute ear infections, and passed newborn hearing screening.

#### Stimuli

The same sentence stimuli were used as in Experiment 1. The visual displays were designed to assess the contribution of rigid and non-rigid movement to perceptual matching by independently testing the effects of global head motion and local face cues, respectively [39]. Rigid-only head motion was presented to one group of infants, and non-rigid face-internal movement was presented to the other group (see Movies S3 and S4).

#### Procedure

The same infant procedure described in Experiment 1 was used.

### Results and Discussion

As in Experiment 1, the percentage of time infants spent fixating the matching visual display as proportion of total trial length was used to index the degree to which infants matched auditory visual ID speech in rigid and non-rigid conditions. Partial eta^2^ is used to report effect size. Again, assumptions of normality were met prior to conducting ANOVAs. The preliminary ANOVAs testing counterbalancing effects were non-significant.

**Figure 3:**
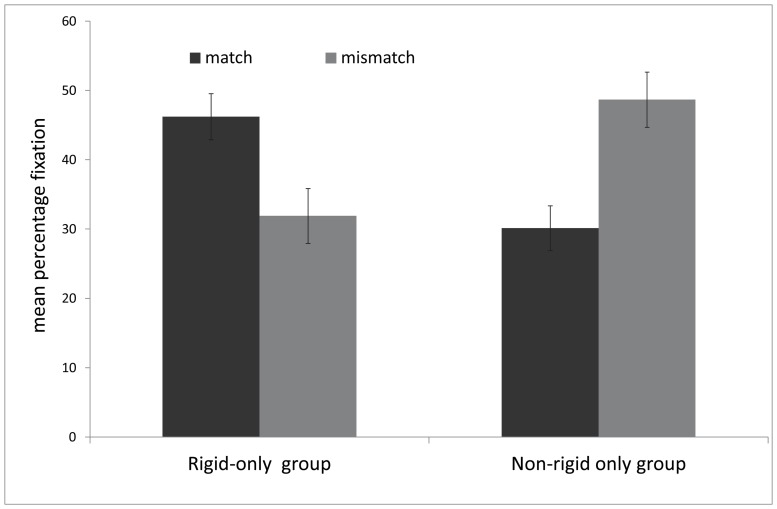
Mean percentage fixation to the matching and mismatching point-line displays in the rigid only and the non-rigid only groups in Experiment 2. Error bars = 1 standard error of the mean.

A 2 × 3 × (2) ANOVA testing group (rigid vs. non-rigid) and syllable difference (2, 3, or 4 syllables) between participants and trial (matching vs. mismatching) within participants revealed no significant main effects for trial, group or syllable number (*p*s>0.17). However, there was significant trial x group interaction *F*(1,34) = 7.1, *p*<0.01; *η_p_^2^* = 0.18. No other interactions were significant (*p*s>0.32). In the rigid group, infants spent a greater percentage of trial duration looking to the matching (*M* = 46.23%) than the mismatching display (*M* = 31.9%) while in the non-rigid group infants preferred the mismatching (*M* = 48.7%) over the matching display (*M* = 30.1%) (see Figure 3). Raw looking time scores (seconds) were also analysed using ANOVA with the same results. Binomial tests were significant. In the rigid condition 17 of the 20 infants looked longer to the matching than the mismatching display (*binomial p* = .001) and in the non-rigid condition 14 of the 20 infants preferred the mismatching over the matching display (*binomial p* = .04).

Importantly the results revealed that in the rigid group, infants looked longer to the matched head and voice, whereas the non-rigid group showed a preference for the mismatched face and voice (see Figure 3). The results of the rigid-only condition were similar to the results of Experiment 1, indicating that infants were able to match the seen and heard speech based on global prosodic information without the benefit of articulatory information. The absence of mouth motion in the rigid-only condition strongly suggests that these results are not based on low-level synchrony detection driven by the perception of synchronous energy onsets and offsets (see [41]), but by the correlated acoustic and visual dynamics of a talking head.

## General Discussion

The results show that 8-month-old infants preferentially fixate matching over mismatching talking faces whether the auditory speech is presented as full spectrum or low-pass filtered at 400 Hz (Experiment 1). Infants did this from the kinematics of point-line displays of talking faces/heads and without the benefit of fine phonetic detail in the visual displays or phonetic detail in the auditory speech, suggesting that performance was based upon matching coarse-grain prosodic information from both modalities. Moreover, the same preference for matched displays was found when rigid head motion alone was presented with facial motion held constant (Experiment 2), indicating that detecting an auditory-visual speech match does not necessarily depend on the temporal relationship of lip movements and voice in the two modalities. Indeed, when restricted to talking faces displaying lip/jaw motion, the infants in the non-rigid group preferred the mismatched to the matched display. Eight-month-olds could clearly discriminate the non-rigid visual displays, and were evidently sensitive to correlated auditory and visual attributes of local (syllable) rhythmic cues in connected speech. Nonetheless, as we predicted, infants were more sensitive when dependent on global prosodic cues than the auditory-visual correlates occurring at the local rhythmic level.

Overall, the results support the proposition that infants perceptually prioritize intersensory signals that share common properties, particularly the prosodic gestures from head movement. The paradox is, however, that with face-only information, infants prefer incongruent syllable sequences across auditory and visual modalities. Typically infants can recognise auditory and visual correspondences in single syllable stimuli [8], [9], [10] or the same sentence presented in and out of synchrony [15], [41]. However, when presented with two point-line talking faces and a sentence, they preferentially attend to the mismatched face. It is unclear why infants looked longer to the mismatched lips and voice displays in the non-rigid condition. It is possible that with a lack of rigid head motion, the motion of the joined segments (e.g., mouth, jaw) were no longer perceived as a gestalt talking head, and the separation of motion acted to increase the difficulty of the non-rigid matching task. It seems that adults can identify speech better when non-rigid motion is accompanied by the appropriate rigid movements than when it is not [42]. Even for adults, it seems that the visual prosody signaled by head motion matters.

Compared to the non-rigid stimuli, the rigid-only stimuli provided a task with low cognitive demand, as it was more visually expressive, making it easier for infants to match the acoustic and visual correlates of sentences when the dominant feature was prosodic variation, and not smaller, more rapidly realised sequences of localised syllable size events. Not only does prosody evolve more slowly over time, but it is a relatively primitive mode of communication, and as such easier to perceive and to be decoded non-linguistically. Thus, it is in the non-rigid condition, that the effects of uncoupling rigid and non-rigid elements into separate motions are most marked, and shown in the infants' attention being drawn to the mismatching display. We suggest that our findings implicate firstly, the rate of prosodic change and secondly, the type of prosody. Prosody can be either linguistic as it conveys linguistic distinctions, such as word boundaries, syntactic structure and lexical meaning; or it can be emotional, where it serves to mark intent or attitude involved in the emotional or social aspects of language.

One possibility is that this significant looking preference to the unexpected pairing represents an instance of a shift from a familiarity to a novelty preference [43]. That is, typically infants will prefer to fixate on stimuli that are familiar to them. This preference also operates with cross-modal stimuli, i.e., infants tend to fixate a visual stimulus that matches an auditory one [44]. In the current study, we suggest that familiarity for the infants is defined at the level of rigid head motion for the visual stimuli and rhythm (prosody) for the auditory ones. This is because, for infants these properties are the most salient; exaggerated rigid head motion characterizes infant-directed speech [26] as does exaggerated prosody for auditory speech. However, when rigid head motion is removed and face-only motion presented, the familiarity of the visual stimulus is reduced. In this case, a preference for the more novel of the cross-modal pairs (mismatch) may arise. This tendency to pay attention to a mismatch between face (mouth) motion and speech when the salient head motion signal has been removed may be because infants between 4 and 8 months tend to pay more attention to the mouth region when viewing AV speech stimuli [45]. That is, finding an AV mismatch may be particularly compelling. Whatever is the correct interpretation of the unexpected looking preference, it is clear that this shows that infants can detect the mismatch between non-rigid face motion and speech.

Our findings for Experiment 2 in which infants match global prosody cues (slowly fluctuating rigid head motion plus auditory change) and mismatch local syllable cues (more rapid non-rigid motion and auditory change), give rise to two questions. First, when do infants acquire the ability to match auditory syllable sequences to the congruent talking face without the benefit of rigid motion? To ensure that adults could perform the task, we tested 24 adults using the same stimuli and experimental set up. Adults were found to easily perceive the match in non-rigid condition and indeed performed better in the non-rigid than the rigid condition. In this study, we asked adults (i) to judge which of the two visual displays matched the auditory sentence; and (ii) how confident they were with their choice. Adults correctly identified the matching face in both rigid and non-rigid conditions, and were more confident with their non-rigid than rigid choice (*p* = .01).

For 8-month-old infants, sensitivity to visual prosody conveyed by rigid motion is well developed, but sensitivity to locally produced syllable sequences, is subject to developmental improvement, and should emerge at a later age. By what age is unclear but it would be expected that this should occur in the months following, and perhaps co-occur with the development of language-specific speech perception. Second, by what mechanism do infants detect congruence between auditory and visual displays? It has been suggested that infants' detection of redundancy is based on intersensory responsiveness to temporal synchrony, and that this emerges first, followed by the emergence of responsiveness to duration, rate, and rhythm [46]. It seems that 10- to 11-month-olds cannot detect visual prosody based on temporal synchrony. Blossom and Morgan found infants could only match face/head and voice (with the mouth was obscured) when different sentences were used, but not when temporal synchrony was disrupted [16]. Therefore the mechanism for detecting congruency between auditory and visual prosody may involve perceiving amodal relations that include multiple cues, not just temporal synchrony but also relating to differences in rhythm and rate. That is, infants rely on a combination of cues. Central to this we suggest that the primary cue is the slower rate of global prosody over local rhythm.

The capacity to perceive the prosodic alignment of auditory and visual components of speech in this study suggests that infants use the voice and head kinematics of prosody to extract information about the structure of speech. One of the basic requirements for learning language is to segment fluent speech into meaningful units, initially clauses and phrases and then words. The development of such abilities may evolve from an initial ability to parse the wave form using coarse grain prosodic features carried at the global prosodic level across modalities. Furthermore, such capacity might assist infants in establishing a perception-production link - one that starts at the global level of intonation in rigid head motion, and then incorporating the rhythmic motion of the mouth and jaw to gradually develop into something more fine-grained that involves phonetic distinctions in continuous speech. The future direction of this work is to examine the developmental trajectory of auditory visual speech perception. In sum, the evidence is accumulating for a sophistication in the perception of auditory-visual speech information during the first year of postnatal life - at the level of speech segments [7], [46], [47]; and at the level of prosody in continuous speech, another cornerstone in the scaffold that underpins speech development.

## Supporting Information

Movie S1 **Side by side displays of sentence pairs for full spectrum condition, labeled ‘normal'.**
(AVI)

Movie S2 **Side by side displays of sentence pairs for low pass filtered condition, labeled ‘prosody'.**
(AVI)

Movie S3 **Side by side displays of sentence pairs for rigid condition, labeled ‘rigid'.**
(AVI)

Movie S4 **Side by side displays of sentence pairs for non-rigid condition, labeled ‘non-rigid'.**
(AVI)
